# Resection of Accessory Parotid Gland Tumors: A Multidisciplinary Feat and Review of Literature

**DOI:** 10.7759/cureus.34945

**Published:** 2023-02-13

**Authors:** Mohammad Hamza, Gautam Anand

**Affiliations:** 1 Department of General Surgery, All India Institute of Medical Sciences, Patna, IND; 2 Department of General Surgery, Lady Hardinge Medical College, New Delhi, IND

**Keywords:** transoral incision, cheek incision, standard parotidectomy incision, pleomorphic adenoma, accessory parotid lobe tumor

## Abstract

Accessory parotid lobe tumors are very rare. For any mass occupying the mid-cheek, tumors of the accessory parotid gland should be strongly suspected. These tumors are properly evaluated using ultrasonography, computed tomography, and fine needle aspiration cytology. Different surgical approaches are mentioned in the literature for the resection of these parotid lesions. These surgical approaches are often accompanied by the extracapsular dissection of the tumor. The extracapsular dissection of the tumor is an adequate technique to resect tumors of the accessory lobe of the parotid.

## Introduction

The accessory parotid lobe is located indistinctly from the parotid gland [1]. The parotid gland is located in the retromandibular fossa while the accessory parotid lobe lies anteriorly to it over the masseter muscle. Any mass arising from the accessory lobe can present as a mid-cheek mass. Like parotid gland masses, accessory lobe masses are usually benign and make up to 1-8% of all parotid tumors [2]. But the incidence of malignant masses is higher in the accessory lobe up to 26-50% [3]. These tumors are usually painless, gradual in onset, and slow growing. They are located near the distal branches of the facial nerve. On proper evaluation by means of radiological studies and histopathological examination, these tumors are often diagnosed. Different surgical approaches are there in the literature for the resection of these parotid lesions. A standard parotidectomy incision, direct cheek incision, and transoral incision are the used surgical approach. These surgical approaches are often accompanied by the extracapsular dissection of the tumor. The standard parotidectomy incision or modified Blair’s incision is the most commonly recommended technique [3]. But due to the sparsity of accessory parotid lobe tumors, these techniques are often debated. We have presented our experience of surgical management of these tumors and reviewed the literature.

## Case presentation

**Figure 1:**
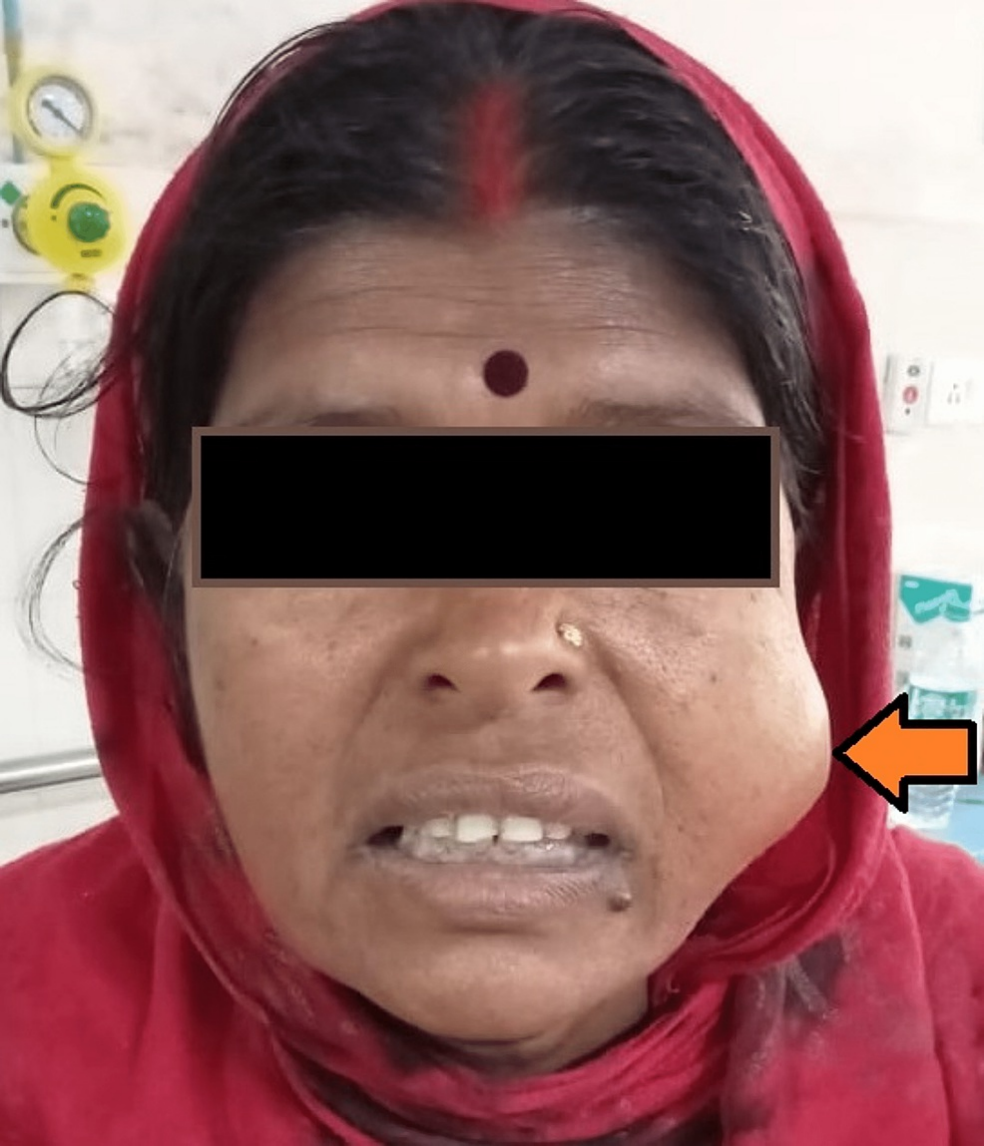
A well-defined mid-cheek swelling of the left side of the face.

**Figure 2:**
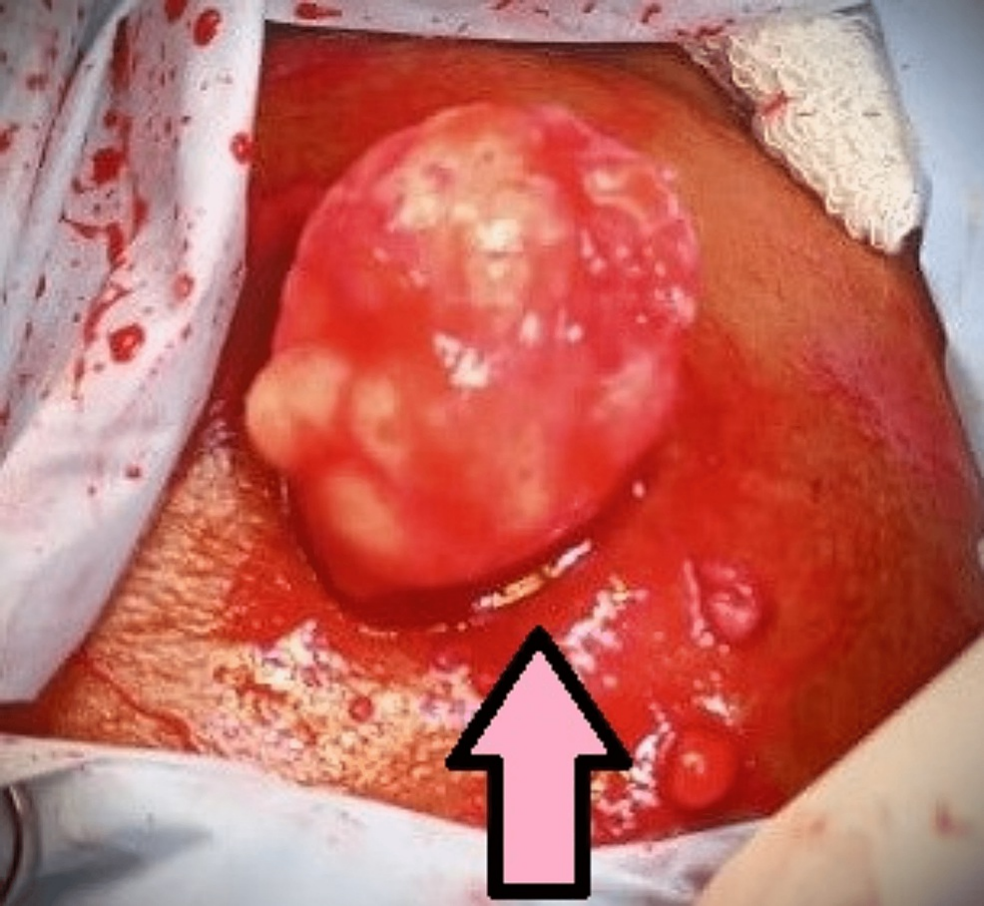
Intraoperative finding of left accessory lobe parotid tumor through left cheek incision.

**Figure 3:**
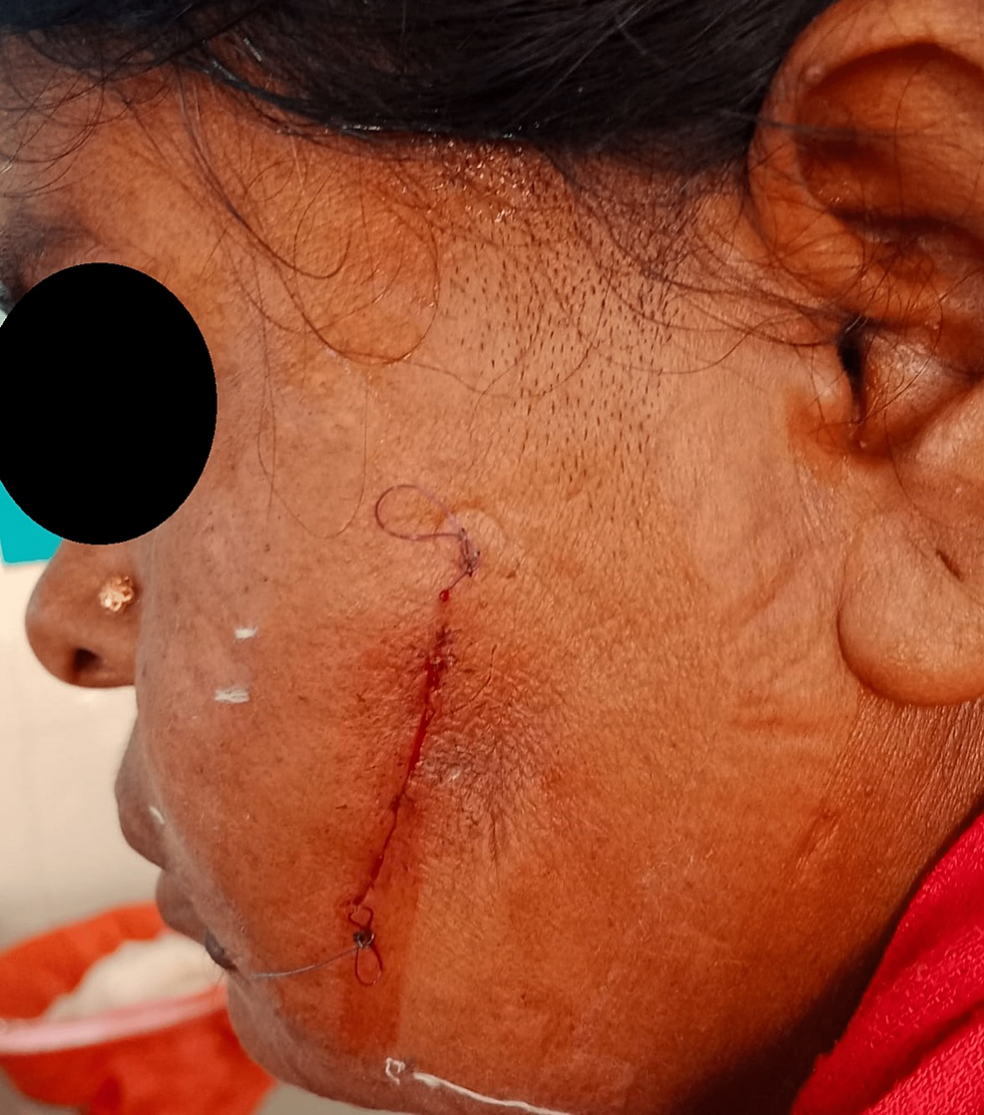
Post-operative status of the patient with a cheek scar and with no post-op facial paresis.

**Figure 4:**
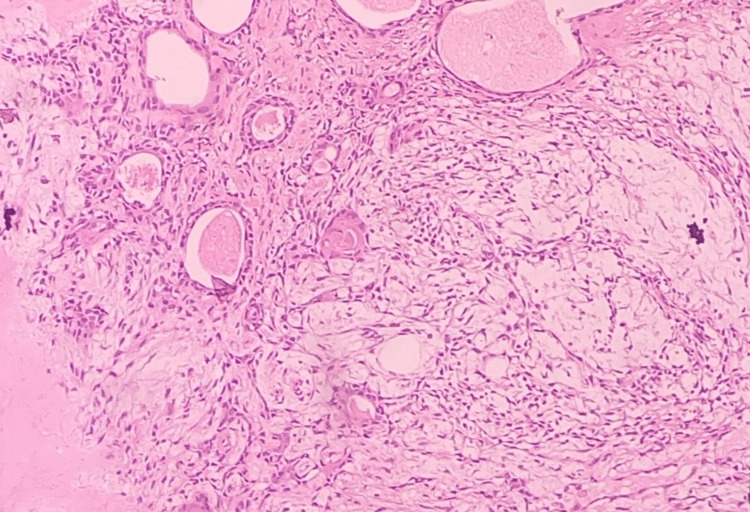
H&E stained (100x) specimen of accessory parotid gland tumors showing mixed epithelial, myoepithelial and stromal cells.

A 50-year-old lady complained of having a mid-cheek mass in the left side of her face for four years. The swelling was painless, gradual in onset and slowly growing. There was no increase in size of the swelling while eating or chewing. On examination, the swelling was 3x3cm in size, non-tender, firm in consistency, mobile in both axes and not fixed to the underlying structures (Figure 1). Ultrasonography of the left cheek was suggestive of a well-defined heteroechoic nodular solid lesion in the subcutaneous plane of the left cheek lying over the masseter muscle of size 2.5x3.2cm with no evidence of calcification and necrosis and with mild internal vascularity. Fine needle aspiration cytology (FNAC) was done, which was suggestive of pleomorphic adenoma. The patient was prepared for the surgery with due consent for chances of injury to the distal branches of the facial nerve. A small mid-cheek incision of size 4.5cm was given over the tumor along the natural skin crease. The tumor was visualized and was dissected free from the underlying masseter muscle, sparing the distal facial nerve branches (Figure 2). An extracapsular dissection of the tumor with resection was done and sent for histopathological examination. The patient tolerated the procedure well. Postoperatively, the facial nerve was intact with no clinical evidence of facial paresis and with minimal facial scar (Figure 3). Histopathology confirmed the tumor to be a pleomorphic adenoma (Figure 4).

## Discussion

Tumors of the accessory parotid lobe are extremely rare. These tumors are usually anterior to the retromandibular fossa lying over the masseter muscle [4]. They present as a mid-cheek mass having minimal symptoms [5]. These masses can lie between the buccal and zygomatic branches of the facial nerve as the course of the facial nerve is complex [6]. The differential diagnosis includes benign or malignant accessory parotid gland tumors, parotid gland cysts, neural tumors, metastatic disease, Kimura disease, and vascular lesions such as hemangioma. Pleomorphic adenoma is the most common tumor arising from the accessory parotid followed by mucoepidermoid carcinoma [7]. These mid-cheek masses should be properly evaluated i.e by a careful physical examination, radiological studies and FNAC. Ultrasonography is increasingly chosen as the initial investigation of choice in salivary gland imaging. It can identify the organ of origin, the parotid duct and detect any duct dilatation due to calculi or soft tissue calcification. Contrast-enhanced Computed Tomography is the most useful tool for assessing internal anatomy, distinguishing cystic from solid tumors, and for its internal vascularity, while magnetic resonance imaging (MRI) is often considered superior to computed tomography for better soft tissue delineation. Therefore it is preferred sometimes over computed tomography in the evaluation of salivary gland tumors. Lastly, FNAC is the final procedure for a definitive histological diagnosis whether benign or malignant which can decide the surgical approach [8]. There are different surgical approaches by which accessory lobe tumors can be resected. These approaches can be a standard parotidectomy incision, direct cheek incision and transoral incision. These surgical approaches are often accompanied by the extracapsular dissection of the tumor. Extracapsular dissection is an adequate technique in resection of accessory lobe parotid tumors [9,10]. The standard parotidectomy incision or modified Blair’s incision is the most commonly recommended technique, which enables good exposure of the tumor with a better margin of resection and minimizes functional and cosmetic deformities [11]. There is less incidence of injury to the facial nerve branches. In cheek incision, the tumors are accessed through a direct incision over the tumor in the mid-cheek region of the face along the natural skin crease. The tumor is then dissected carefully from the masseter muscle and the distal branches of the facial nerve. Johnson and Spiro reported only a 7% incidence of temporary facial palsy with modified Blair’s incision while there was a 40% incidence of facial nerve damage with a cheek incision [12]. Temporary facial nerve palsy associated with modified Blair’s incision had better nerve recovery than cheek incision. The transoral approach involves the excision of the tumors that are located anteriorly and are easily bimanually palpable. It avoids the facial scar and there is direct visualization of the parotid duct. If the tumor is located anteriorly and lies over the masseter muscle, it is best accessed by cheek incision or transoral incision. But, if the tumor is located behind the masseter muscle, it is best managed by standard parotidectomy incision. However, like cheek incision, transoral incision also carries a higher risk of injury to the facial nerve [13].

We have attempted to focus on practical considerations of the various surgical approaches in decision-making before surgery. We have observed that the standard parotidectomy incision with facial nerve trunk exposure and dissection of its peripheral branches is excessive, it has sometimes resulted in unnecessary injury to the branches. We have observed that cheek incision limits the area of dissection and decreases tumor implantation. Cheek incision approaches are quicker as it involves less dissection. Newberry et al. mentioned routinely recognizing the distal facial nerve branches before dissecting the tumor from its bed in the case series [14]. However, we observed that while dissecting the tumor, the facial nerve branches if encountered should be carefully identified and preserved. It will prevent undue exposure of the peripheral nerves and their damage. If a tumor is malignant on FNAC, a standard parotidectomy incision is preferred to ensure an oncologically safe surgical margin. We have reviewed five articles and a total of 46 cases from the literature (Table 1). But due to the sparsity of accessory parotid lobe tumors, these techniques are often debated, which entails further studies.

**Table 1:** Clinical, surgical approach and pathological features of the reported cases of accessory parotid gland tumors PA: Pleomorphic Adenoma, MA: Monomorphic Adenoma, SCC: Squamous Cell Carcinoma, MEC: Mucoepidermoid Carcinoma, BCL: B Cell Lymphoma, BCAd: Basal Cell Adenocarcinoma, PC: Parotid Cyst, nAPG: normal Accessory Parotid Gland, CEA: Carcinoma Expleomorphic Adenoma, LEC: Lymphoepithiloma-like Carcinoma, SP: Standard Parotidectomy Incision, TO: Transoral Incision.

Author	N	F/M	Mean age(yrs)	Size(cm)	Surgical approach	Histology	Mean follow up
Singh et al. [3]	8	4/4	41(18-51)	NA	4 SP, 3 TO, 1 cervical incision	7 PA + 1 MEC	9(1-11)
Choi et al. [4]	8	3/5	35(14-47)	4.92	8 SP	4PA + LEC + 1 hemangioma + 1 PC + 1 BCAd	44(6-120)
Klotz et al. [5]	9	2/7	56(28-90)	4.05	4 SP, 3 observations, 1 radiation, 1 revision maxillectomy	4PA + 1 nAPG + 1 SCC + 1 metastatic SCC + 1 lymph node + 1 muscle hypertrophy	NA
Lin et al. [6]	8	5/3	54(36-70)	4.58	7 SP + 1 observation	2 PA + 2 MA + 1 undifferentiated carcinoma + 1 CEA + 1BCAd + 1PC	27.8
Newberry et al. [14]	13	7/6	47(14-65)	4.32	13 SP	4 PA, 1 MA2 lymphadenitis + 2MEC, 2 BCL, 1 adenocarcinoma +1 myofibrosarcoma	39.6

## Conclusions

Any mid-cheek mass not typical of a parotid lump but close to the parotid region should be treated with a high degree of suspicion of accessory lobe parotid tumors. Due to higher incidence of malignant tumors arising from the accessory lobe, these lumps should be carefully evaluated prior to definitive treatment. Ultrasonography and ultrasonography-guided FNAC should be done. MRI can further delineate the anatomy. Although a standard parotidectomy incision approach is appropriate in most cases, other direct approaches may also be considered depending on the location of the tumor.
